# Supplementary material for: From data compilation to model validation: comparing three ecosystem models of the Tasman and Golden Bays, New Zealand
Source: PeerJ. 2021 Jul 19;9:e11712. doi: 10.7717/peerj.11712 (PMC8415284; doi:10.7717/peerj.11712)
Supplement: Supplemental Information 1 [file peerj-09-11712-s001.pdf]

Supplementary A: ROMS model varibles

Temperature and salinity from ROMS model, used to force the Tasman and Golden Bays Atlantis model.

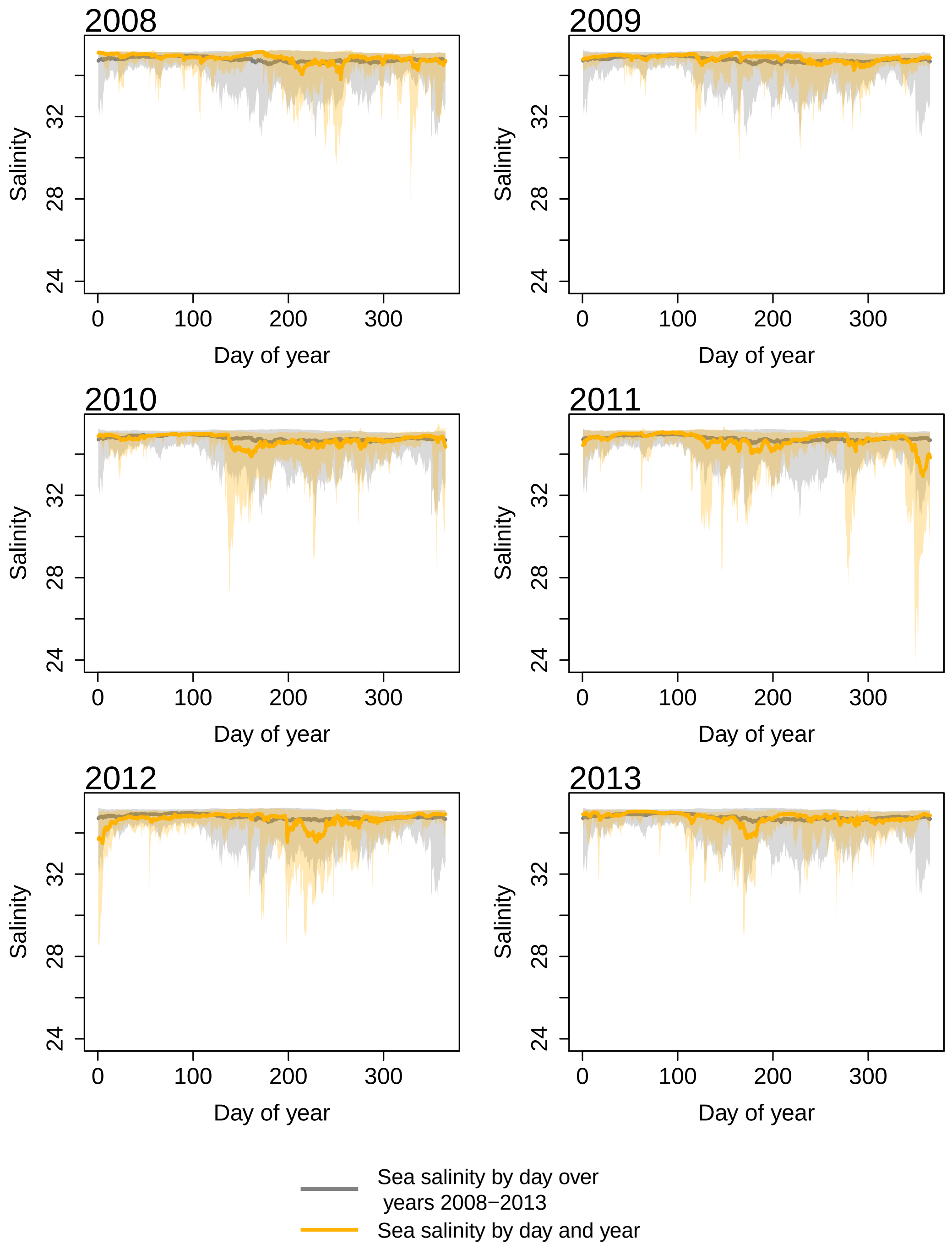

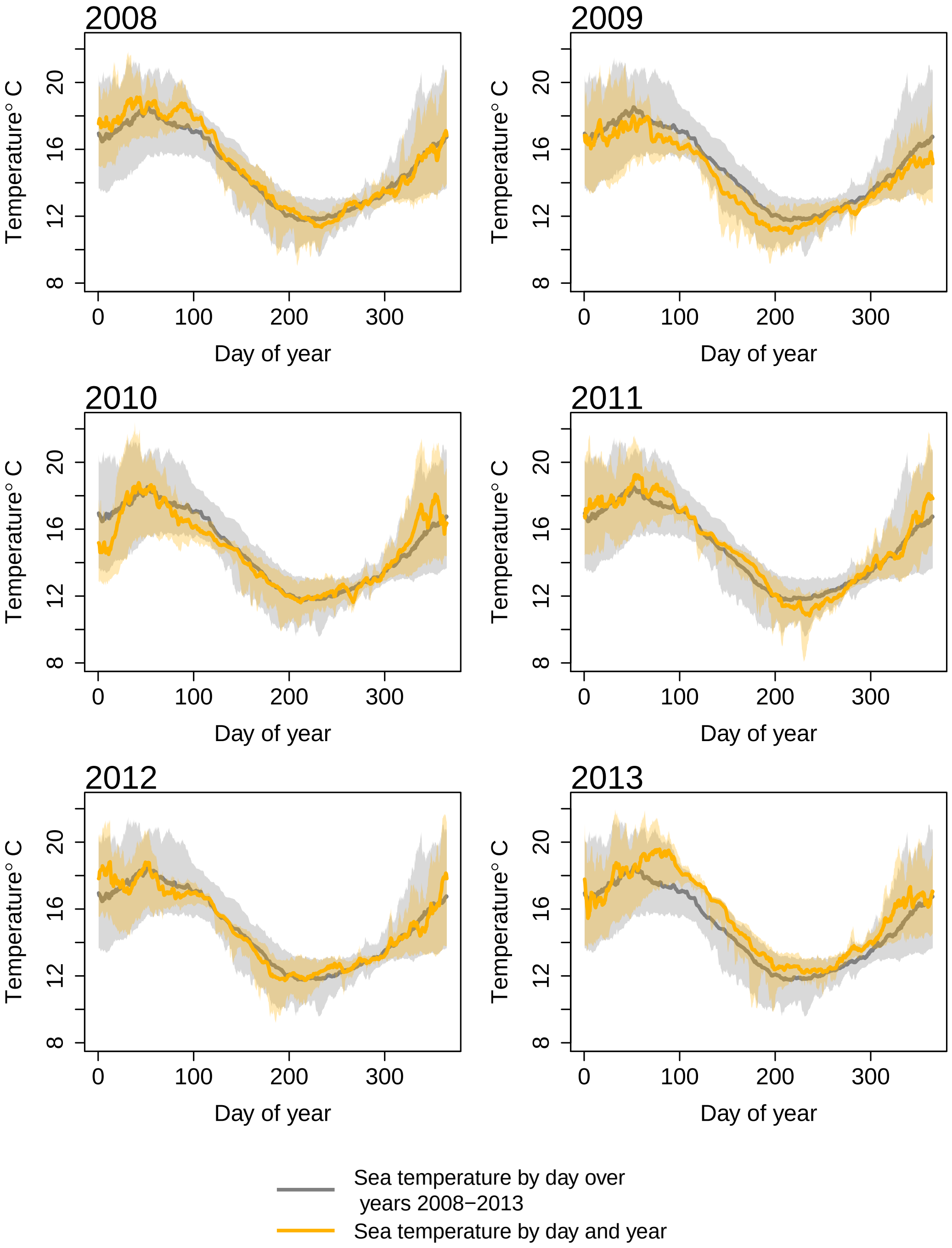
2008
2009
2010
2011
2012
2013
Temperature° C
8
12
16
20
0
100
200
300
Day of year
Sea temperature by day over
years 2008−2013
Sea temperature by day and year

Supplementary B: Initial conditions and biological parameters for species groups

Initial biomasses for each species group were estimated using a single species stochastic stock assessment model, CASAL (Bull et al., 2012). Biomass estimates for the entire Tasman and Golden Bays were derived by using known biological parameters and a catch history to project back from an absolute abundance estimate in 2002. Values of relative abundance were available for most species groups from trawl surveys conducted annually from 1992 to 2013 (see Stevenson and MacGibbon (2018)). For each survey, these abundance estimates were converted to absolute values using trawl catchability quotients (specific to each group) derived by our expert opinion, as fisheries scientists with experience dating back more than 30 years. Estimated absolute abundance for each group in 2002 (the midpoint of the survey series) was taken as the mean from all the survey estimates. For each species group, the initial biomass estimate was distributed across polygons in proportion to the survey series estimates (i.e., the mean proportion of total biomass by polygon over the survey series). The distribution of biomass by depth layer in each polygon was derived using our expert opinion.

For fish species or species groups that were not sampled, or were very poorly sampled, by the survey series (i.e., snapper, kahawai, pelagic and reef fishes), initial biomasses were derived using expert opinion on their likely abundance relative to species groups that had been estimated by modelling.

Initial biomasses of the commercially exploited bivalve groups were derived primarily as maximum estimates of biomass from any survey from the Tasman and Golden Bays (Fisheries New Zealand, 2020). For cephalopods, the mean biomass of arrow squid from the 1992–2013 research trawl survey series (Stevenson and MacGibbon, 2018), with a small additional allocation for octopus, was assumed to be the initial biomass. For the remaining invertebrate groups, initial biomass were set based on our expert opinion on their likely abundance relative to species groups that had been otherwise estimated. Initial biomasses of invertebrate groups were allocated to each cell based on distribution information from all available research surveys (bivalves and cephalopods), or on expert opinion on their likely distribution and density (other groups).

Initial biomass of seabirds was estimated to be 13.2 t, derived as 50 000 individuals each weighing 0.26 kg. The number of individuals was the maximum estimate of shore birds reported by Schuckard and Melville (2013) in surveys of the top of the South Island throughout the 2000s. For pinnipeds (fur seals), biomass in 1900 was likely to be low relative to estimates from recent years,

owing to heavy exploitation of the species in the 1800s (Baird, 2011). An initial biomass of 8 t was estimated, derived as 100 adults with an average weight of 80 kg. Pup production in recent years in Tasman and Golden Bays is estimated to be about 500 annually (Baird, 2011), so is indicative of a population of at least 500 adult females.

For age-structured groups, initial biomass estimates were assigned to age-classes using estimates of instantaneous natural mortality (M). Initial average weights at age were calculated using Von Bertalanffy growth and length-weight conversion parameters. Values used for these parameters are in Table 1. Weights at age were split into reserve and structural components using ratio $R_N : S_N = 2.5 : 1$. This allows for an individual's body mass to decrease by approximately 70% before starving, which is within the 60–80% range suggested by Broekhuizen et al. (1994).

Table 1: Biological parameters assumed for age-structured species groups. VB, von Bertalanffy; M, instantaneous natural mortality rate; h, steepness value for the Beverton-Holt stock recruitment relationship. Length-weight parameters are: $W = aL^b$ (weight W in g, length L in cm). Where Reference is 'Trawl db' some data have been derived from the NIWA trawl survey database (see Mackay (2000)). Species group matches 'Name' in Tables **??** and **??** and are without punctuation.

**Species group**	**VB Growth**			**Length-weight**		**M**	**h**	**Reference**
	**Linf (cm)**	**K**	T_0	a	b			
Barracouta	85.2	0.298	-0.45	0.00572	2.9706	0.3		Fisheries New Zealand (2020)
Cephalopod	35	2.4	0	0.029	3		0.8	Fisheries New Zealand (2020)
Demersal fish	78.4	0.14	-0.66	0.0103	3.1376	0.23	0.8	Manning and Sutton (2007)
Elasmobranch Invert	151.8	0.096	-0.78	0.03324	2.8779	0.3	0.3	Francis et al. (2004)
Elasmobranch Pisc	244.2	0.12	-1.76	0.00911	3.08	0.23	0.3	FishBase (Froese and Pauly, 2000)
Flatfish	48.7	0.51	-0.1	0.03846	2.6584	1.1	0.8	Colman (1978)
Red gurnard	43	0.39	-0.66	0.00608	3.1491	0.31	0.8	Sutton (1997)
Invert comm Herb						0.14	0.8	Fisheries New Zealand (2020)
Invert comm Scav						0.12	0.8	Fisheries New Zealand (2020)
Kahawai	54.2	0.3	0.25	0.0103	3.14	0.18	0.8	Drummond and Wilson (1993)
Leatherjacket	27.9	0.8	-0.05	0.027	2.903	0.5	0.8	Visconti et al. (2018)
Mackerels	36	0.3	-0.65	0.028	2.84	0.18	0.7	Horn (1991)
Mesopel fish Invert	65.1	0.225	-0.61	0.016	3.07	0.24	0.8	Horn (2001)
Mussels							0.8	
Dredge oysters	85.4	0.6	0			0.2	0.8	Osborne (1999)
Pelagic fish lge	46.2	0.28	-0.25	0.016	3.064	0.087	0.8	Walsh et al. (1999)

Pelagic fish sml	24.3	0.41	-0.18	0.00458	3.3	0.56	0.7	Paul et al. (2001)
Pinniped						0.16		Taylor et al. (1995)
Red cod	73.9	0.51	0.2	0.01178	2.9268	0.76	0.7	Beentjes (2000)
Reef fish Invert	51.8	0.23	-1.7	0.00609	3.239	0.35	0.8	Paul et al. (2000)
Reef fish Pisc	42.6	0.635	-0.19	0.02169	2.9459	0.4	0.8	Hanchet et al. (2001)
Seabird								
Scallops	14.4	0.4	0			0.46	0.8	Breen (1995)
School shark	165.8	0.104	-2.37	0.00346	3.0708	0.1	0.3	Francis and Mulligan (1998)
Surf clams	4.74	0.4	0.07	0.0002	3.153	0.4	0.8	Osborne (1999)
Snapper	66.7	0.16	-0.11	0.04467	2.793	0.075	0.9	Gilbert and Sullivan (1994)
Spiny dogfish	104.8	0.093	-3.17	0.0013	3.2639	0.2	0.3	Hanchet (1986)
Rig	147.2	0.119	-2.35	0.00477	2.9692	0.25	0.3	Francis and Ó Maolagáin (2000)
Tarakihi	44.7	0.237	-0.62	0.01502	3.0539	0.1	0.8	Stevenson and Horn (2004)

Supplementary C: Simulated biomass by species group from no-fishing model

Simulated biomass from the un-fished model (black line) with 95% confidence intervals based on 20% CVs (Coefficient of Variation) shaded orange by species group.

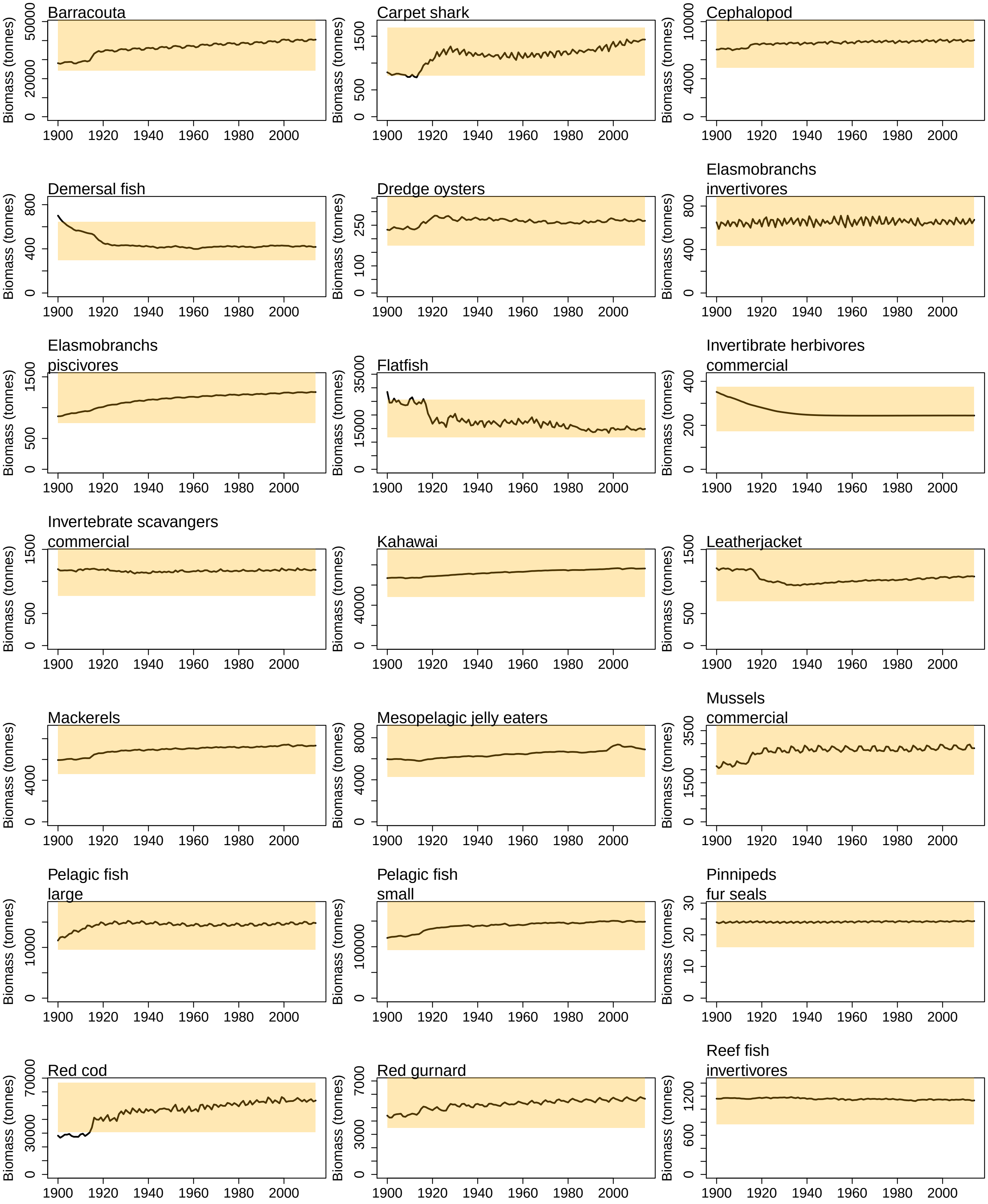
Barracouta
Carpet shark
Cephalopod
Demersal fish
Dredge oysters
Elasmobranchs invertivores
Elasmobranchs piscivores
Flatfish
Invertibrate herbivores commercial
Invertebrate scavangers commercial
Kahawai
Leatherjacket
Mackerels
Mesopelagic jelly eaters
Mussels commercial
Pelagic fish large
Pelagic fish small
Pinnipeds fur seals
Red cod
Red gurnard
Reef fish invertivores
Biomass (tonnes)
1900
1920
1940
1960
1980
2000

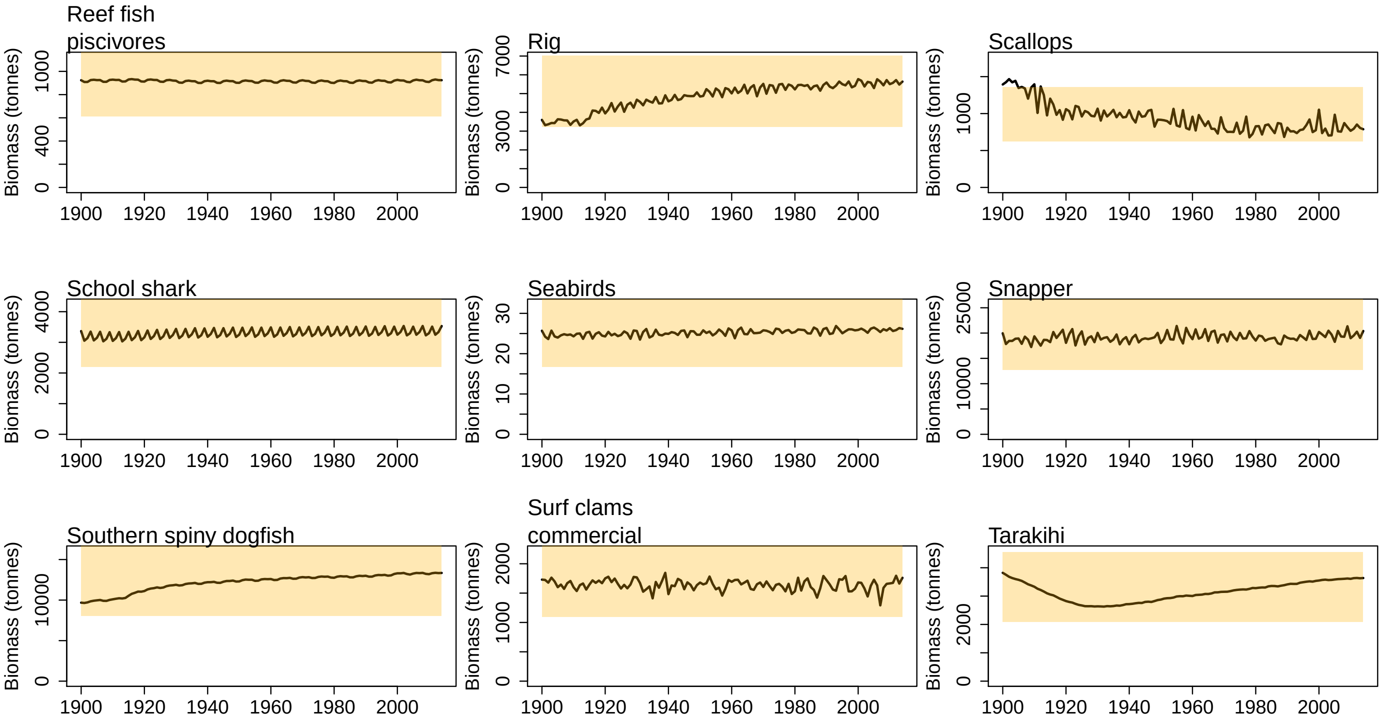
Reef fish piscivores
Rig
Scallops
School shark
Seabirds
Snapper
Southern spiny dogfish
Surf clams commercial
Tarakihi
Biomass (tonnes)
1900
1920
1940
1960
1980
2000

Supplementary D: Size-at-age

Size-at-age using values based on literature (Table 1) (orange lines), from TBGB_SS base non-fished model at equilibrium (blue lines) and from TBGB_AM simulated years 1900–2014 with grey shaded area showing 90% confidence intervals.

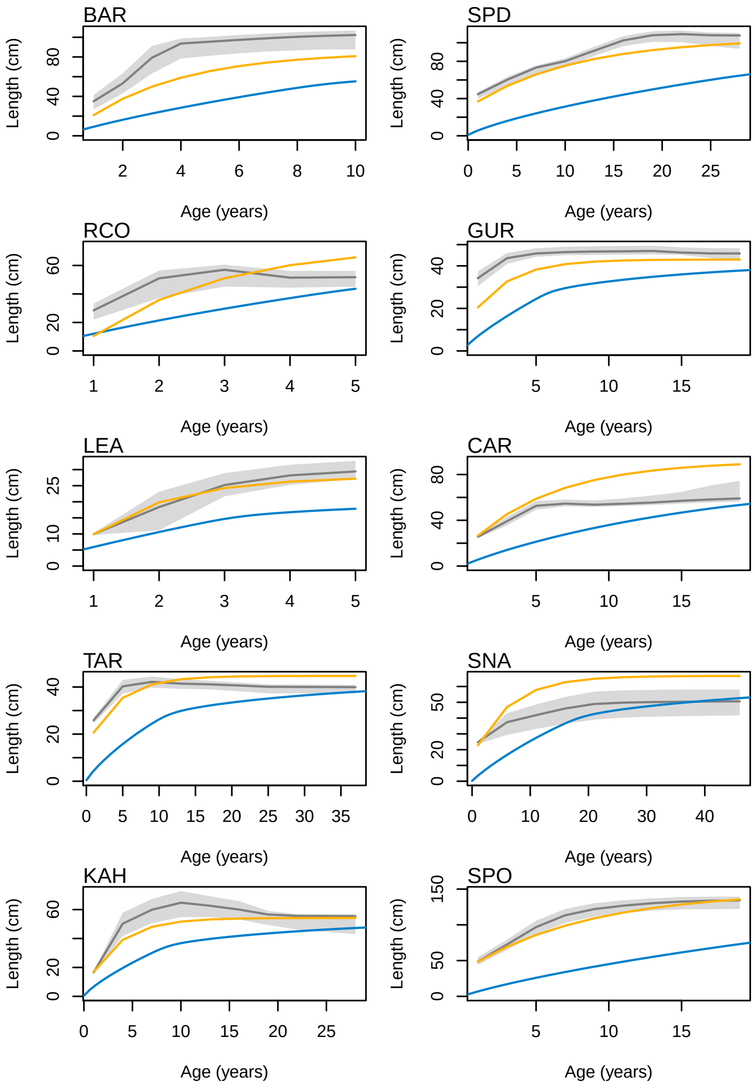

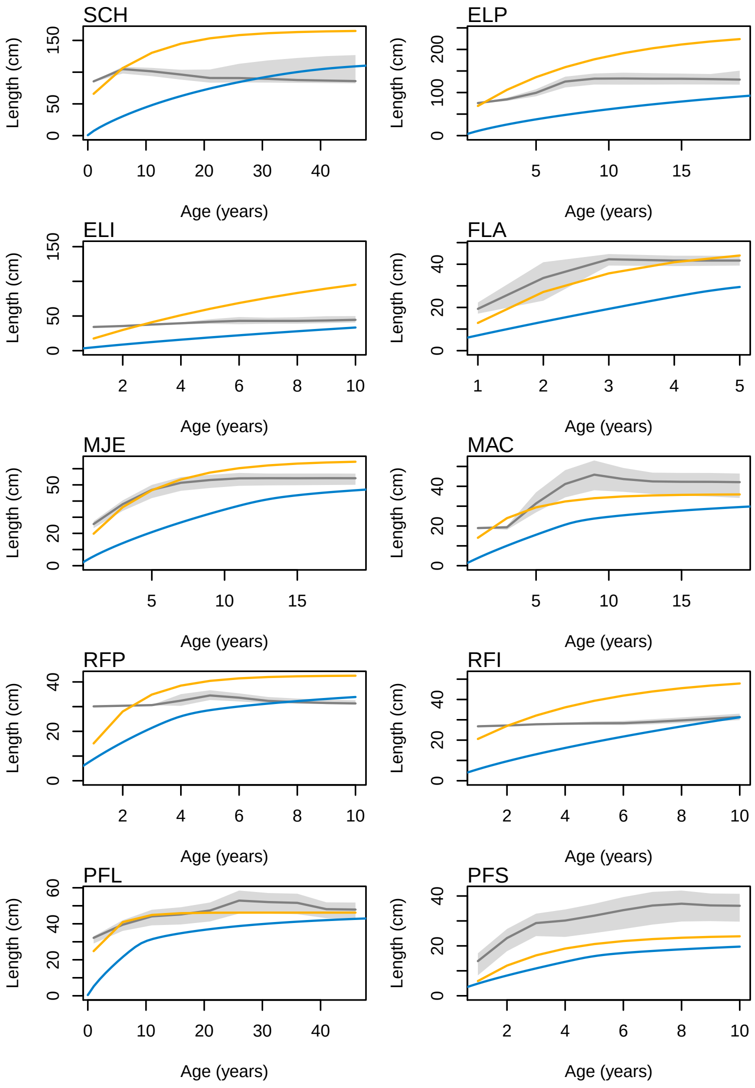

SCH
ELP
ELI
FLA
MJE
MAC
RFP
RFI
PFL
PFS
Length (cm)
Age (years)

Supplementary E: Proportion-at-age

Proportions at age using M based on literature (Table 1) where available (blue lines) and from TBGB_AM simulated years 1900–2014 (boxplots).

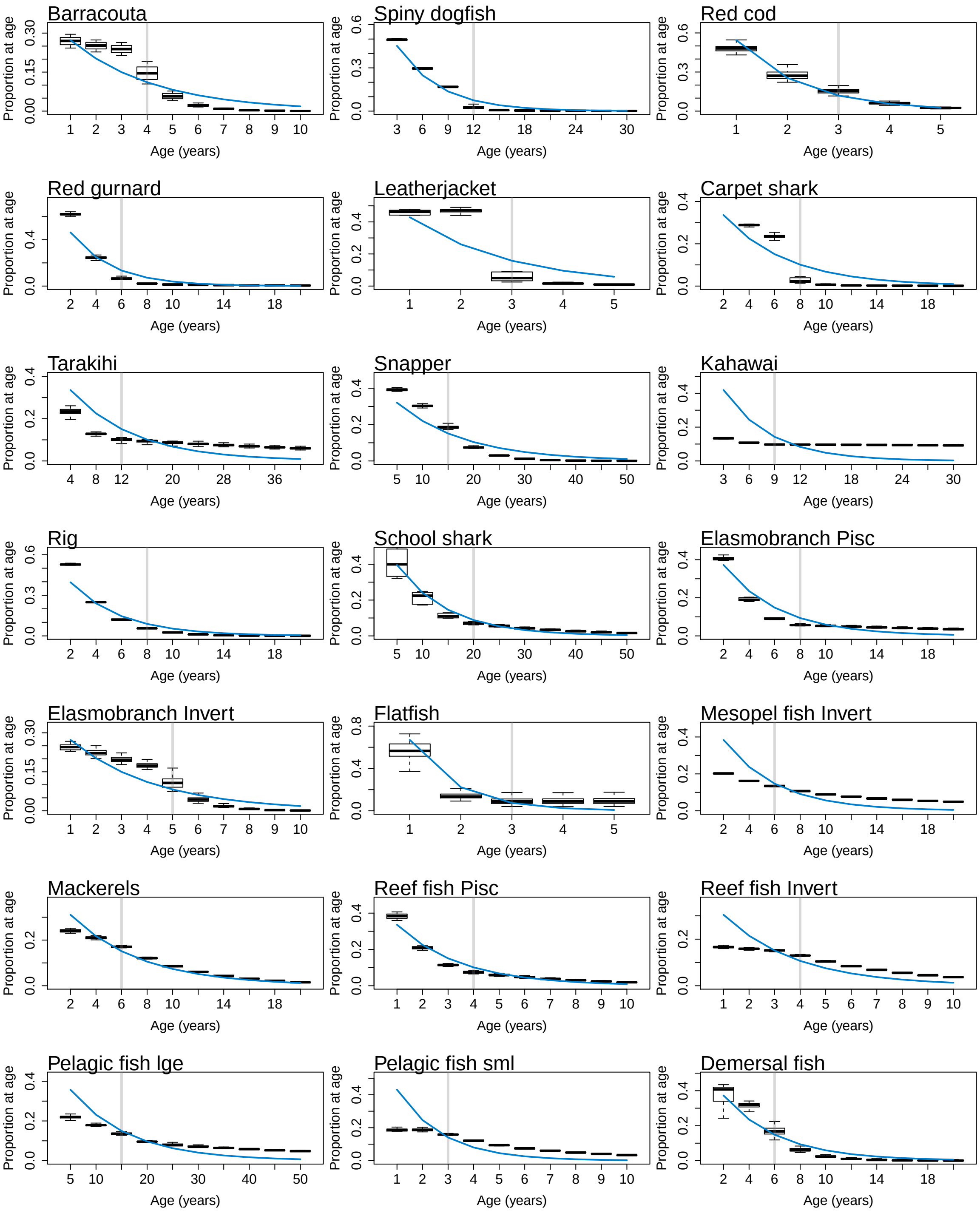

Supplementary F: Data sources for catch histories

Data sources for catch histories used in TBGB_AM, TBGB_EwE and TBTB_SB for 1900–2013.

Years	Data source	Details
1989–2013	Quota Management System (QMS)	'warehou' database administered by Fisheries New Zealand, along with additional data associated with each catch record (i.e., date, fishing method, target species, latitude and longitude)
1983–1988	Fisheries Statistics Unit (FSU)	'new_fsu' database administered by NIWA for Fisheries New Zealand, along with additional data associated with each catch record (i.e., date, fishing method, target species, latitude and longitude)
1974–1982	Fisheries Statistics Unit (FSU)	Catch records from this era were recorded by the Fisheries Statistics Unit of the Ministry of Agriculture and Fisheries, and compiled by Francis and Paul (2013). Catches were available by species, by year, by port of landing (i.e., there was no information relating to month, fishing method, or catch location)
1931–1973	Annual Reports on Fisheries	Annual Reports on Fisheries, compiled by the Marine Department until 1971 and the Ministry of Agriculture and Fisheries until 1973 as a component of their Annual Reports to Parliament, contained estimates of annual catches by species and port of landing, and these were summarised by Francis and Paul (2013). There was no information relating to month, fishing method, or catch location
1900–1930	Annual Reports on Fisheries	Annual Reports on Fisheries, compiled by the Marine Department for the years 1915 to 1930 contained lists of the main species landed by port, estimated landing weight of all species combined (occasionally with some estimates for individual species or species groups), as well as some information on fleet structure (Marine Department Annual Report, 1916–1931).
1900–1915	Back-projected from 1915	The Annual Reports commenced in 1902, but contained little useful quantitative information until 1916. However, catch histories for the model were projected back to 1900, using 1915 as a starting point and assuming a commercial fishery in 1900 that was roughly 10% as productive as in 1915

Supplementary G: Compare TBGB_AM Model responses to fishing to survey biomass estimates

TBGB_AM Model biomass (black line), forced historical fishing (grey bars) and survey biomass estimates (midnightblue pluses).

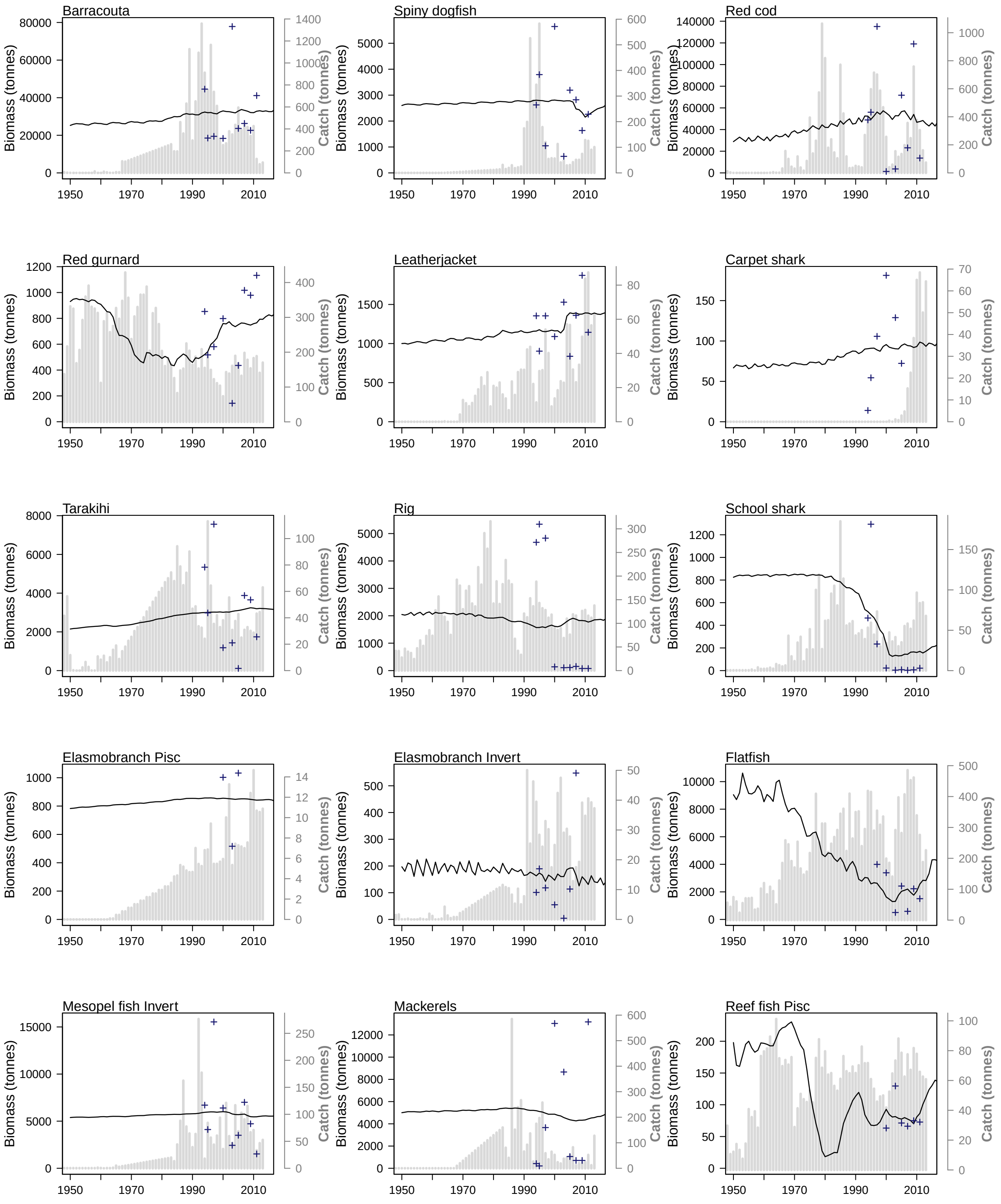

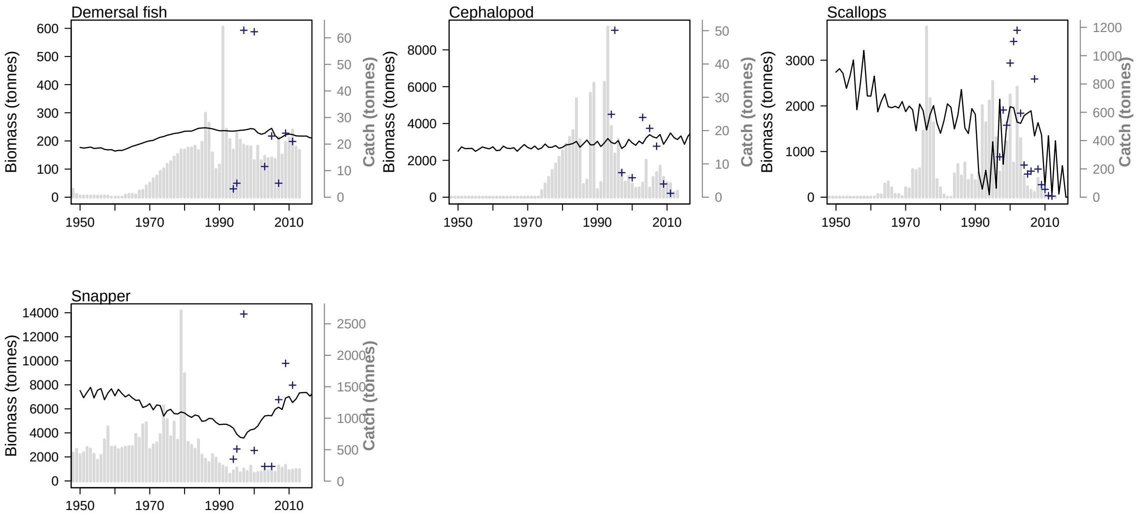
Demersal fish
Cephalopod
Scallops
Snapper
Biomass (tonnes)
Catch (tonnes)
1950
1970
1990
2010

Supplementary H: Diet comparisons

Proportions of prey consumed for each predator and for each base un-fished model: TBGB_AM summarised over 1900–2014 (grey bars); TBGB_EwE summarised over 1959–2014 (orange bars); TBGB_SS at equilibrium (blue bars).

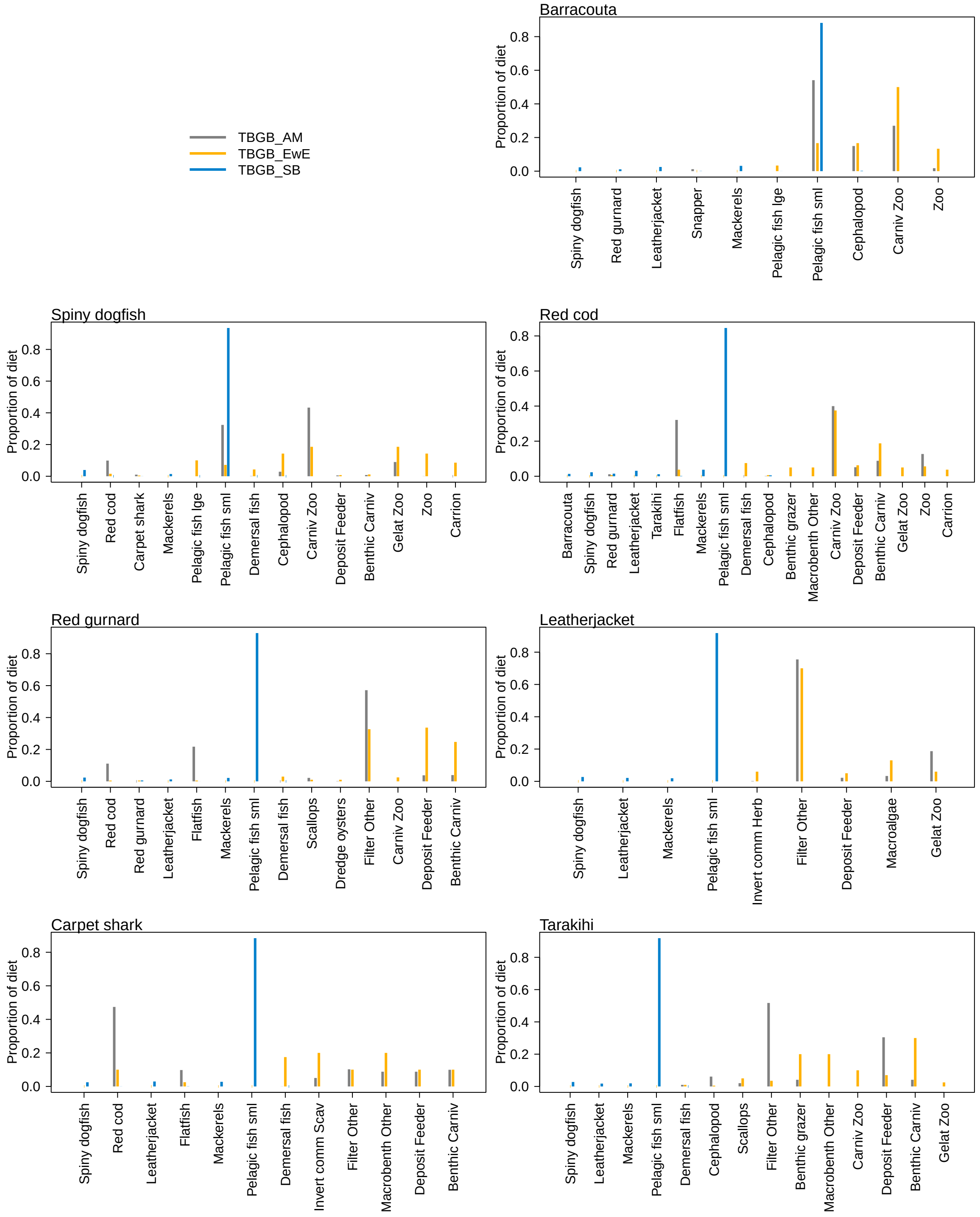

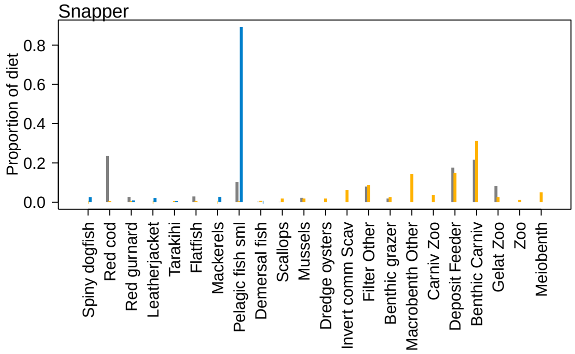
Snapper
Proportion of diet
0.0
0.2
0.4
0.6
0.8
Spiny dogfish
Red cod
Red gurnard
Leatherjacket
Tarakihi
Flatfish
Mackerels
Pelagic fish sml
Demersal fish
Scallops
Mussels
Dredge oysters
Invert comm Scav
Filter Other
Benthic grazer
Macrobenth Other
Carniv Zoo
Deposit Feeder
Benthic Carniv
Gelat Zoo
Zoo
Meiobenth

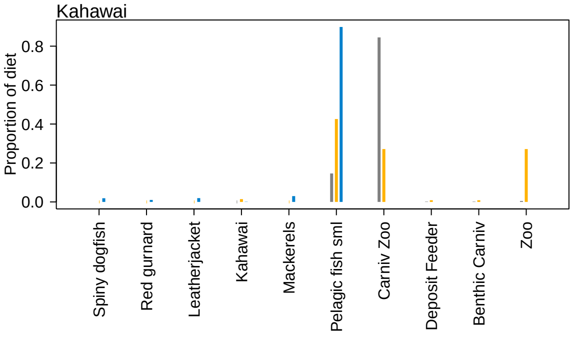
Kahawai
Proportion of diet
0.0
0.2
0.4
0.6
0.8
Spiny dogfish
Red gurnard
Leatherjacket
Kahawai
Mackerels
Pelagic fish sml
Carniv Zoo
Deposit Feeder
Benthic Carniv
Zoo

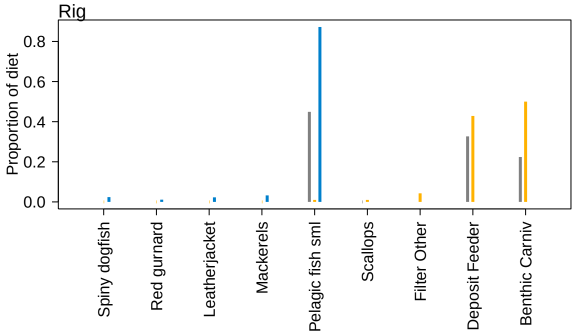
Rig
Proportion of diet
0.0
0.2
0.4
0.6
0.8
Spiny dogfish
Red gurnard
Leatherjacket
Mackerels
Pelagic fish sml
Scallops
Filter Other
Deposit Feeder
Benthic Carniv

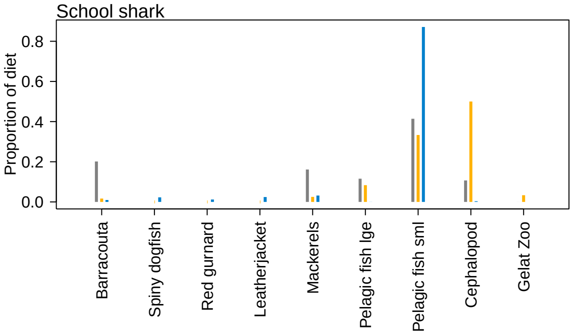
School shark
Proportion of diet
0.0
0.2
0.4
0.6
0.8
Barracouta
Spiny dogfish
Red gurnard
Leatherjacket
Mackerels
Pelagic fish lge
Pelagic fish sml
Cephalopod
Gelat Zoo

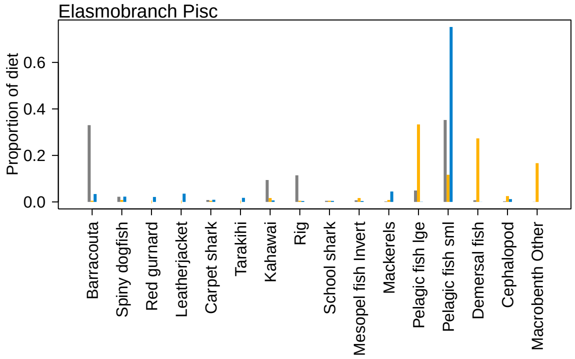
Elasmobranch Pisc
Proportion of diet
0.0
0.2
0.4
0.6
Barracouta
Spiny dogfish
Red gurnard
Leatherjacket
Carpet shark
Tarakihi
Kahawai
Rig
School shark
Mesopel fish Invert
Mackerels
Pelagic fish lge
Pelagic fish sml
Demersal fish
Cephalopod
Macrobenth Other

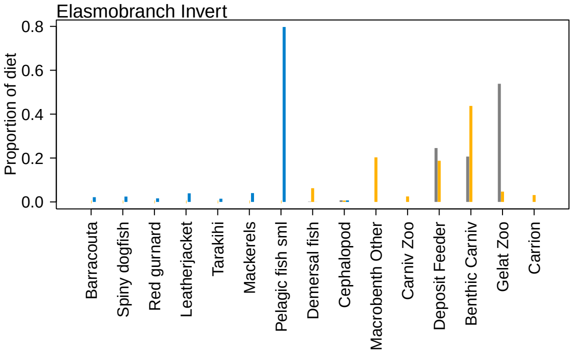
Elasmobranch Invert
Proportion of diet
0.0
0.2
0.4
0.6
0.8
Barracouta
Spiny dogfish
Red gurnard
Leatherjacket
Tarakihi
Mackerels
Pelagic fish sml
Demersal fish
Cephalopod
Macrobenth Other
Carniv Zoo
Deposit Feeder
Benthic Carniv
Gelat Zoo
Carrion

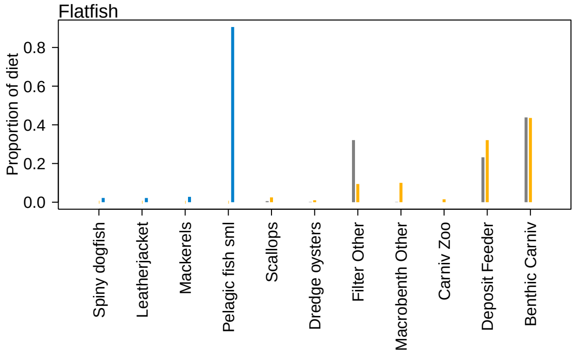
Flatfish
Proportion of diet
0.0
0.2
0.4
0.6
0.8
Spiny dogfish
Leatherjacket
Mackerels
Pelagic fish sml
Scallops
Dredge oysters
Filter Other
Macrobenth Other
Carniv Zoo
Deposit Feeder
Benthic Carniv

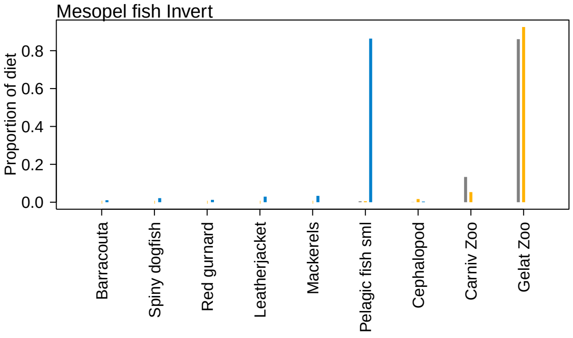
Mesopel fish Invert
Proportion of diet
0.0
0.2
0.4
0.6
0.8
Barracouta
Spiny dogfish
Red gurnard
Leatherjacket
Mackerels
Pelagic fish sml
Cephalopod
Carniv Zoo
Gelat Zoo

78

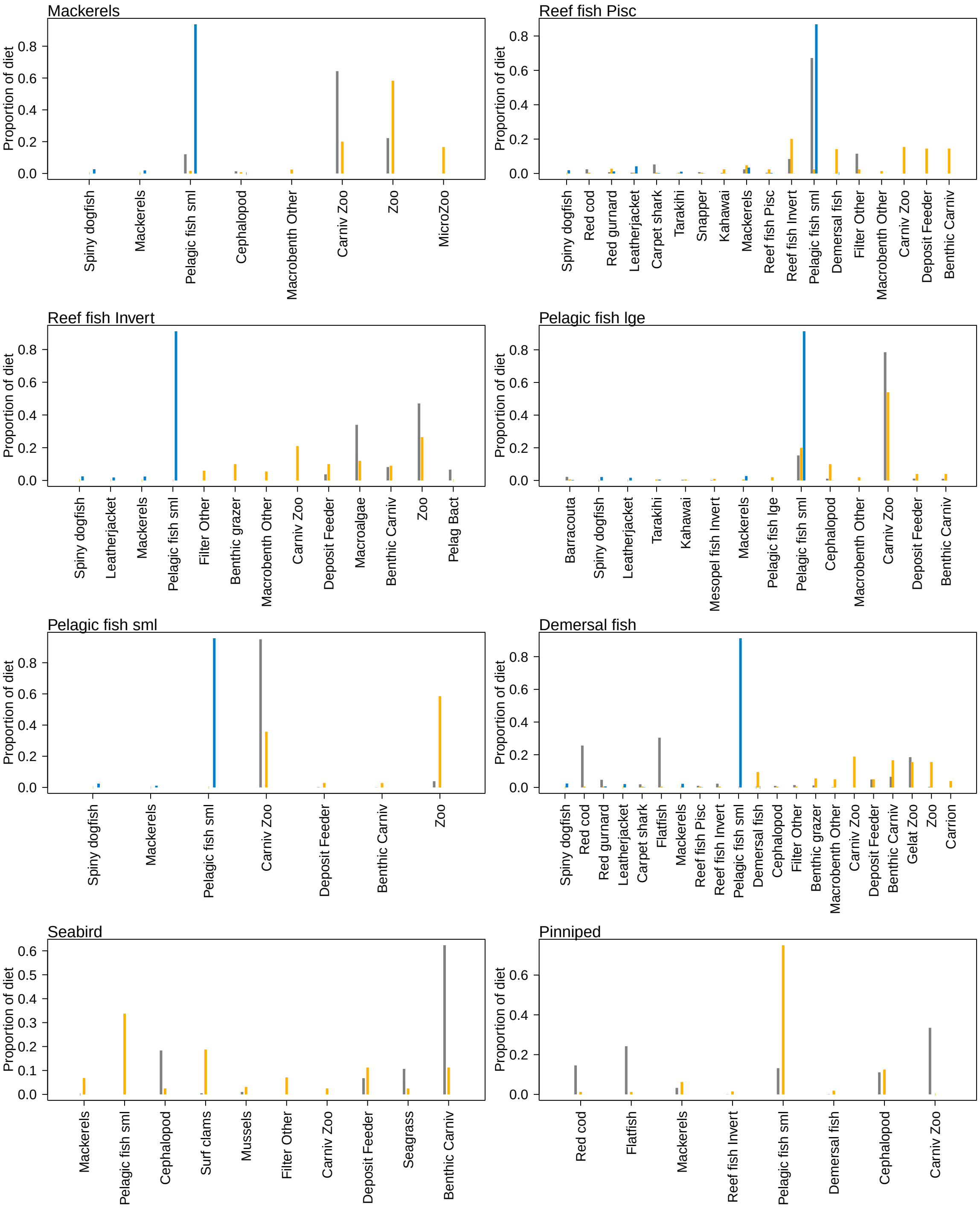
Mackerels
Proportion of diet
0.0 0.2 0.4 0.6 0.8
Spiny dogfish
Mackerels
Pelagic fish sml
Cephalopod
Macrobenth Other
Carniv Zoo
Zoo
MicroZoo
Reef fish Pisc
Proportion of diet
0.0 0.2 0.4 0.6 0.8
Spiny dogfish
Red cod
Red gurnard
Leatherjacket
Carpet shark
Tarakihi
Snapper
Kahawai
Mackerels
Reef fish Pisc
Reef fish Invert
Pelagic fish sml
Demersal fish
Filter Other
Macrobenth Other
Carniv Zoo
Deposit Feeder
Benthic Carniv
Reef fish Invert
Proportion of diet
0.0 0.2 0.4 0.6 0.8
Spiny dogfish
Leatherjacket
Mackerels
Pelagic fish sml
Filter Other
Benthic grazer
Macrobenth Other
Carniv Zoo
Deposit Feeder
Macroalgae
Benthic Carniv
Zoo
Pelag Bact
Pelagic fish lge
Proportion of diet
0.0 0.2 0.4 0.6 0.8
Barracouta
Spiny dogfish
Leatherjacket
Tarakihi
Kahawai
Mesopel fish Invert
Mackerels
Pelagic fish lge
Pelagic fish sml
Cephalopod
Macrobenth Other
Carniv Zoo
Deposit Feeder
Benthic Carniv
Pelagic fish sml
Proportion of diet
0.0 0.2 0.4 0.6 0.8
Spiny dogfish
Mackerels
Pelagic fish sml
Carniv Zoo
Deposit Feeder
Benthic Carniv
Zoo
Demersal fish
Proportion of diet
0.0 0.2 0.4 0.6 0.8
Spiny dogfish
Red cod
Red gurnard
Leatherjacket
Carpet shark
Flatfish
Mackerels
Reef fish Pisc
Reef fish Invert
Pelagic fish sml
Demersal fish
Cephalopod
Filter Other
Benthic grazer
Macrobenth Other
Carniv Zoo
Deposit Feeder
Benthic Carniv
Gelat Zoo
Zoo
Carrion
Seabird
Proportion of diet
0.0 0.1 0.2 0.3 0.4 0.5 0.6
Mackerels
Pelagic fish sml
Cephalopod
Surf clams
Mussels
Filter Other
Carniv Zoo
Deposit Feeder
Seagrass
Benthic Carniv
Pinniped
Proportion of diet
0.0 0.2 0.4 0.6
Red cod
Flatfish
Mackerels
Reef fish Invert
Pelagic fish sml
Demersal fish
Cephalopod
Carniv Zoo

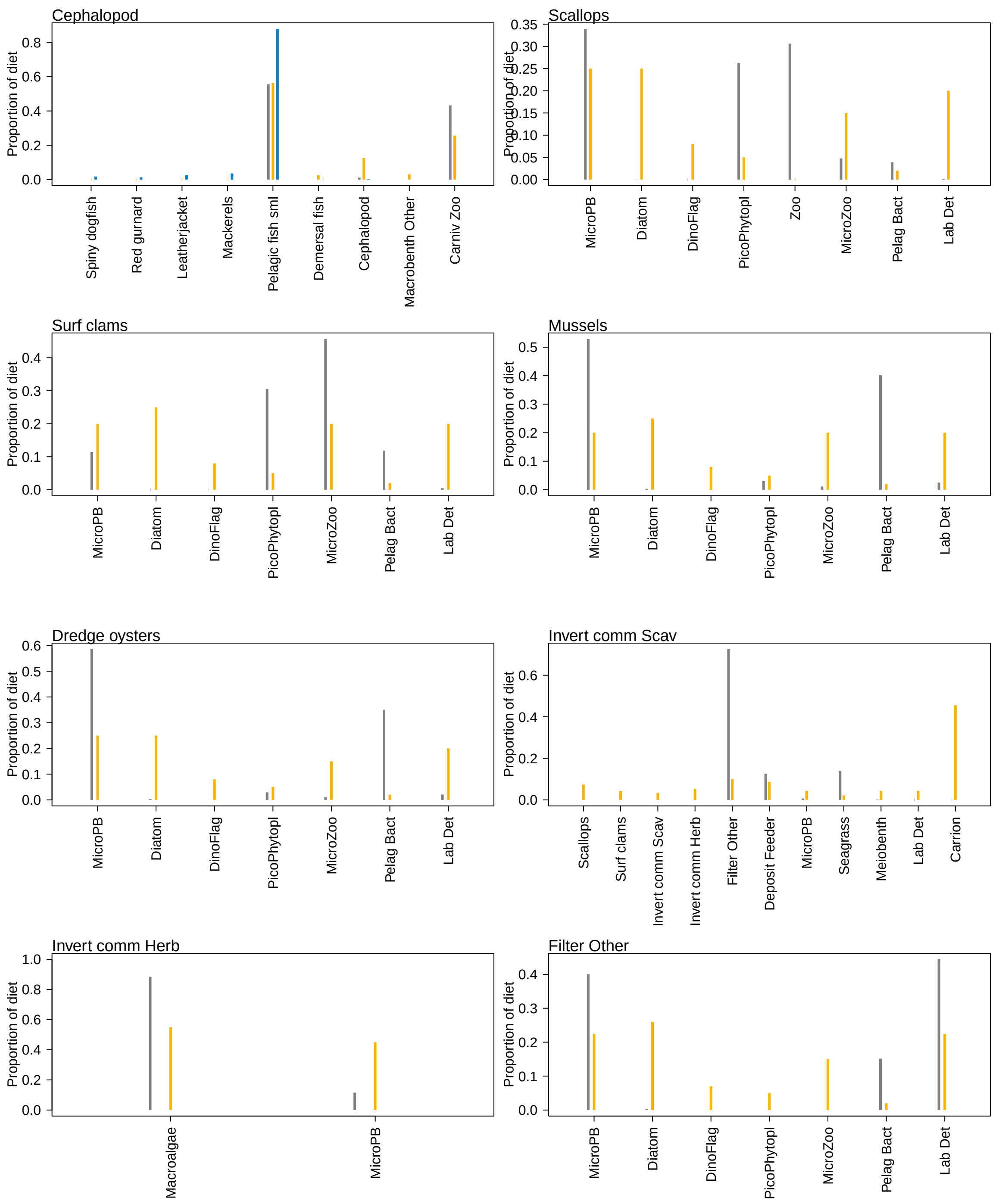
Cephalopod
Proportion of diet
0.0
0.2
0.4
0.6
0.8
Spiny dogfish
Red gurnard
Leatherjacket
Mackerels
Pelagic fish sml
Demersal fish
Cephalopod
Macrobenth Other
Carniv Zoo
Scallops
Proportion of diet
0.00
0.05
0.10
0.15
0.20
0.25
0.30
0.35
MicroPB
Diatom
DinoFlag
PicoPhytopl
Zoo
MicroZoo
Pelag Bact
Lab Det
Surf clams
Proportion of diet
0.0
0.1
0.2
0.3
0.4
MicroPB
Diatom
DinoFlag
PicoPhytopl
MicroZoo
Pelag Bact
Lab Det
Mussels
Proportion of diet
0.0
0.1
0.2
0.3
0.4
0.5
MicroPB
Diatom
DinoFlag
PicoPhytopl
MicroZoo
Pelag Bact
Lab Det
Dredge oysters
Proportion of diet
0.0
0.1
0.2
0.3
0.4
0.5
0.6
MicroPB
Diatom
DinoFlag
PicoPhytopl
MicroZoo
Pelag Bact
Lab Det
Invert comm Scav
Proportion of diet
0.0
0.2
0.4
0.6
Scallops
Surf clams
Invert comm Scav
Invert comm Herb
Filter Other
Deposit Feeder
MicroPB
Seagrass
Meiobenth
Lab Det
Carrion
Invert comm Herb
Proportion of diet
0.0
0.2
0.4
0.6
0.8
1.0
Macroalgae
MicroPB
Filter Other
Proportion of diet
0.0
0.1
0.2
0.3
0.4
MicroPB
Diatom
DinoFlag
PicoPhytopl
MicroZoo
Pelag Bact
Lab Det

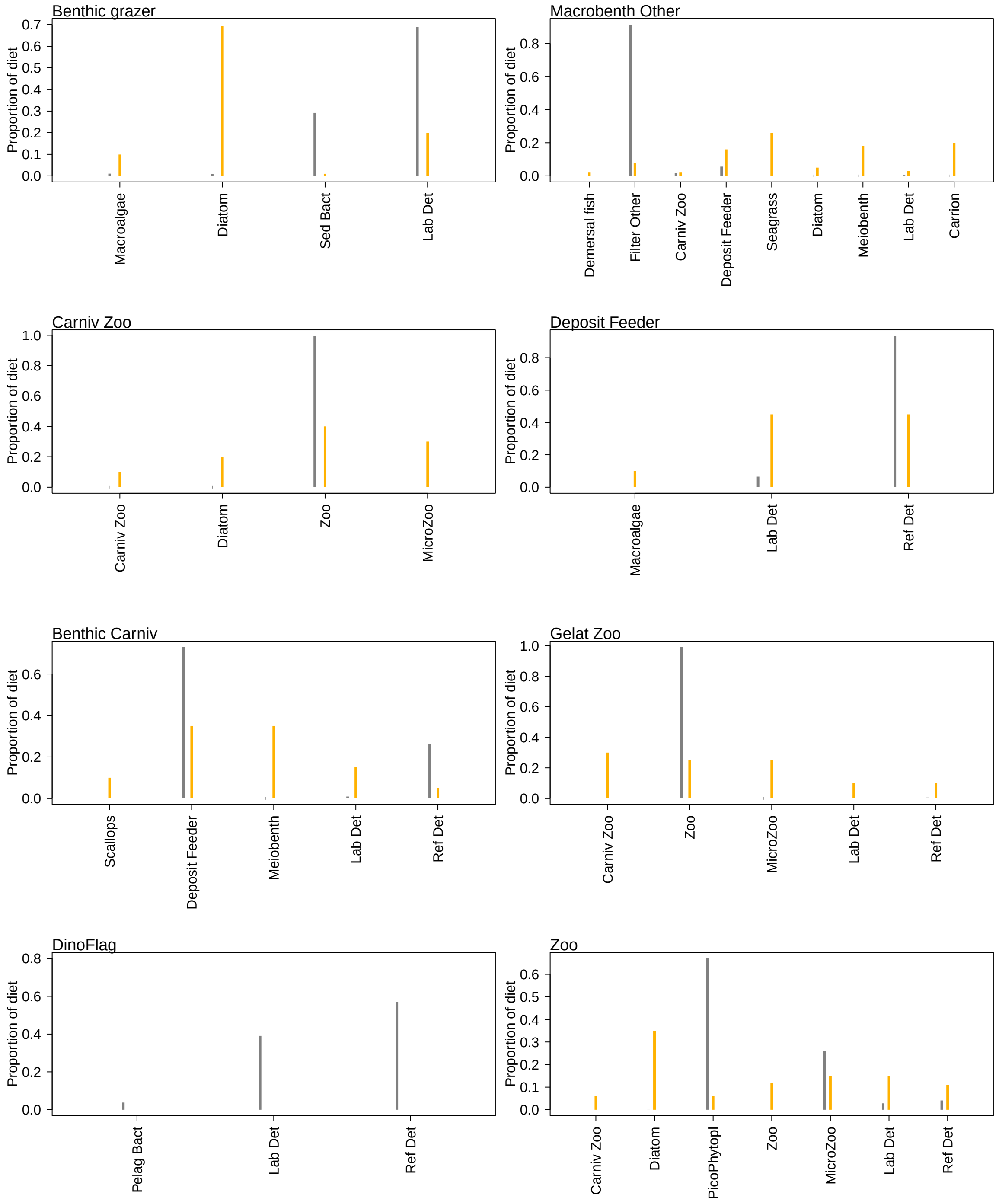
Benthic grazer
Proportion of diet
0.0
0.1
0.2
0.3
0.4
0.5
0.6
0.7
Macroalgae
Diatom
Sed Bact
Lab Det
Macrobenth Other
Proportion of diet
0.0
0.2
0.4
0.6
0.8
Demersal fish
Filter Other
Carniv Zoo
Deposit Feeder
Seagrass
Diatom
Meiobenth
Lab Det
Carrion
Carniv Zoo
Proportion of diet
0.0
0.2
0.4
0.6
0.8
1.0
Carniv Zoo
Diatom
Zoo
MicroZoo
Deposit Feeder
Proportion of diet
0.0
0.2
0.4
0.6
0.8
Macroalgae
Lab Det
Ref Det
Benthic Carniv
Proportion of diet
0.0
0.2
0.4
0.6
Scallops
Deposit Feeder
Meiobenth
Lab Det
Ref Det
Gelat Zoo
Proportion of diet
0.0
0.2
0.4
0.6
0.8
1.0
Carniv Zoo
Zoo
MicroZoo
Lab Det
Ref Det
DinoFlag
Proportion of diet
0.0
0.2
0.4
0.6
0.8
Pelag Bact
Lab Det
Ref Det
Zoo
Proportion of diet
0.0
0.1
0.2
0.3
0.4
0.5
0.6
Carniv Zoo
Diatom
PicoPhytopl
Zoo
MicroZoo
Lab Det
Ref Det

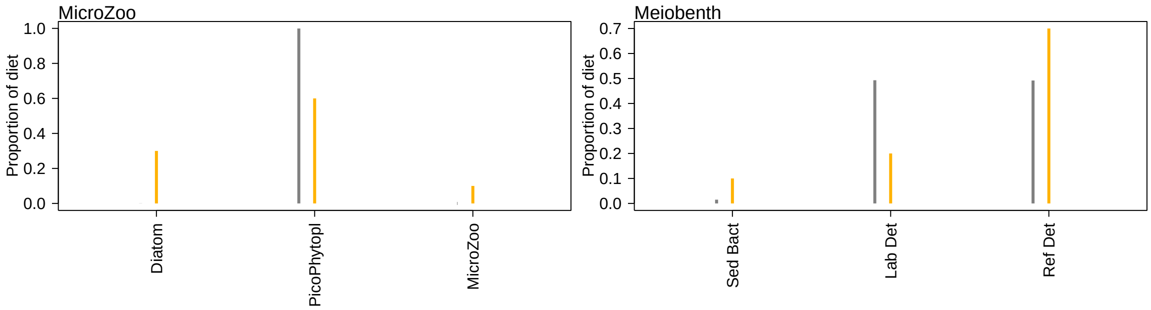
MicroZoo
Proportion of diet
1.0
0.8
0.6
0.4
0.2
0.0
Diatom
PicoPhytopl
MicroZoo
Meiobenth
Proportion of diet
0.7
0.6
0.5
0.4
0.3
0.2
0.1
0.0
Sed Bact
Lab Det
Ref Det

Supplementary I: Model responses to historical fishing

Simulated biomass trajectories for TBGB_AM, TBGB_EwE and TBGB_SS with forced historical catches.

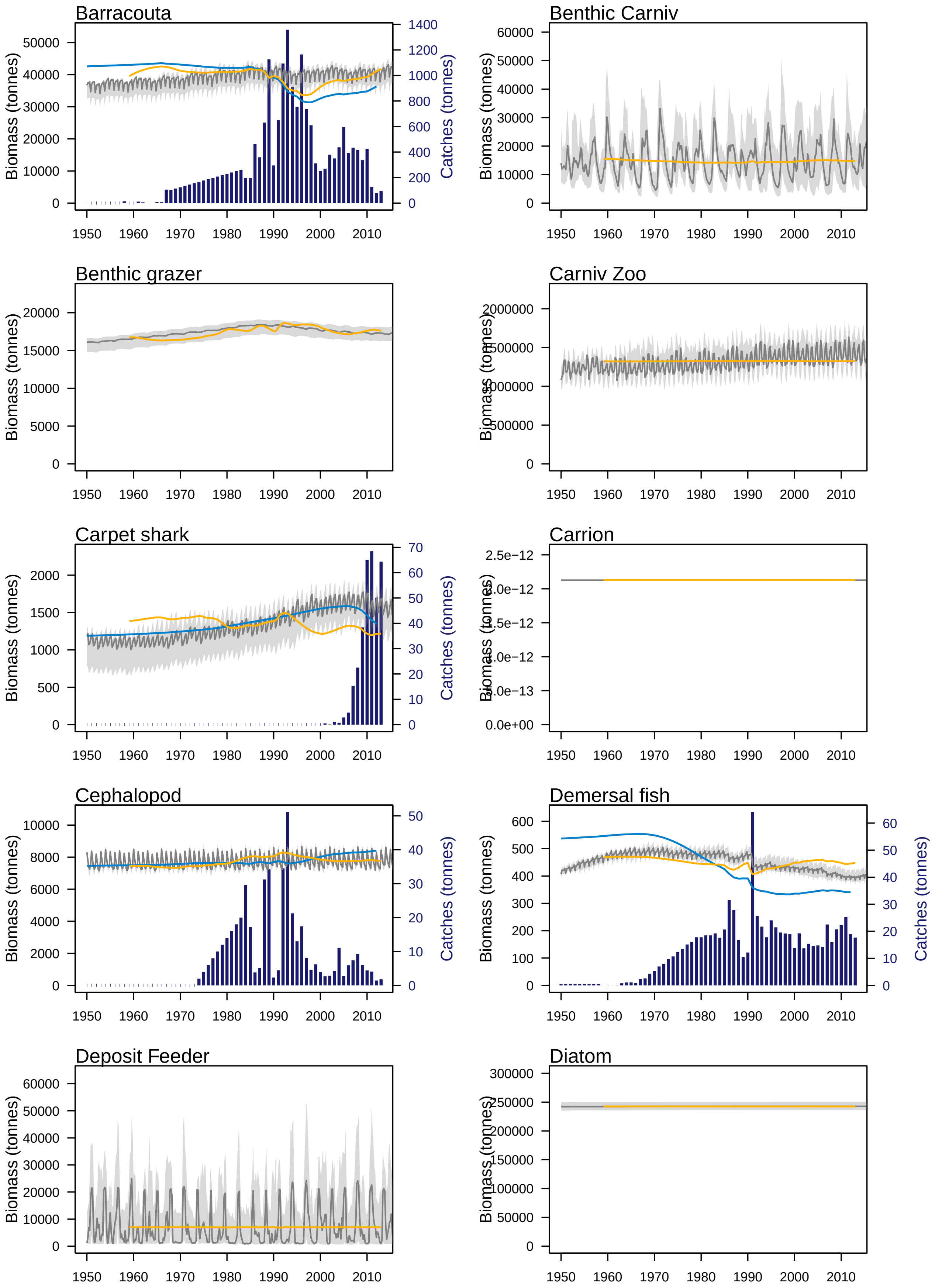
Barracouta
Benthic Carniv
Benthic grazer
Carniv Zoo
Carpet shark
Carrion
Cephalopod
Demersal fish
Deposit Feeder
Diatom
Biomass (tonnes)
Catches (tonnes)
1950 1960 1970 1980 1990 2000 2010

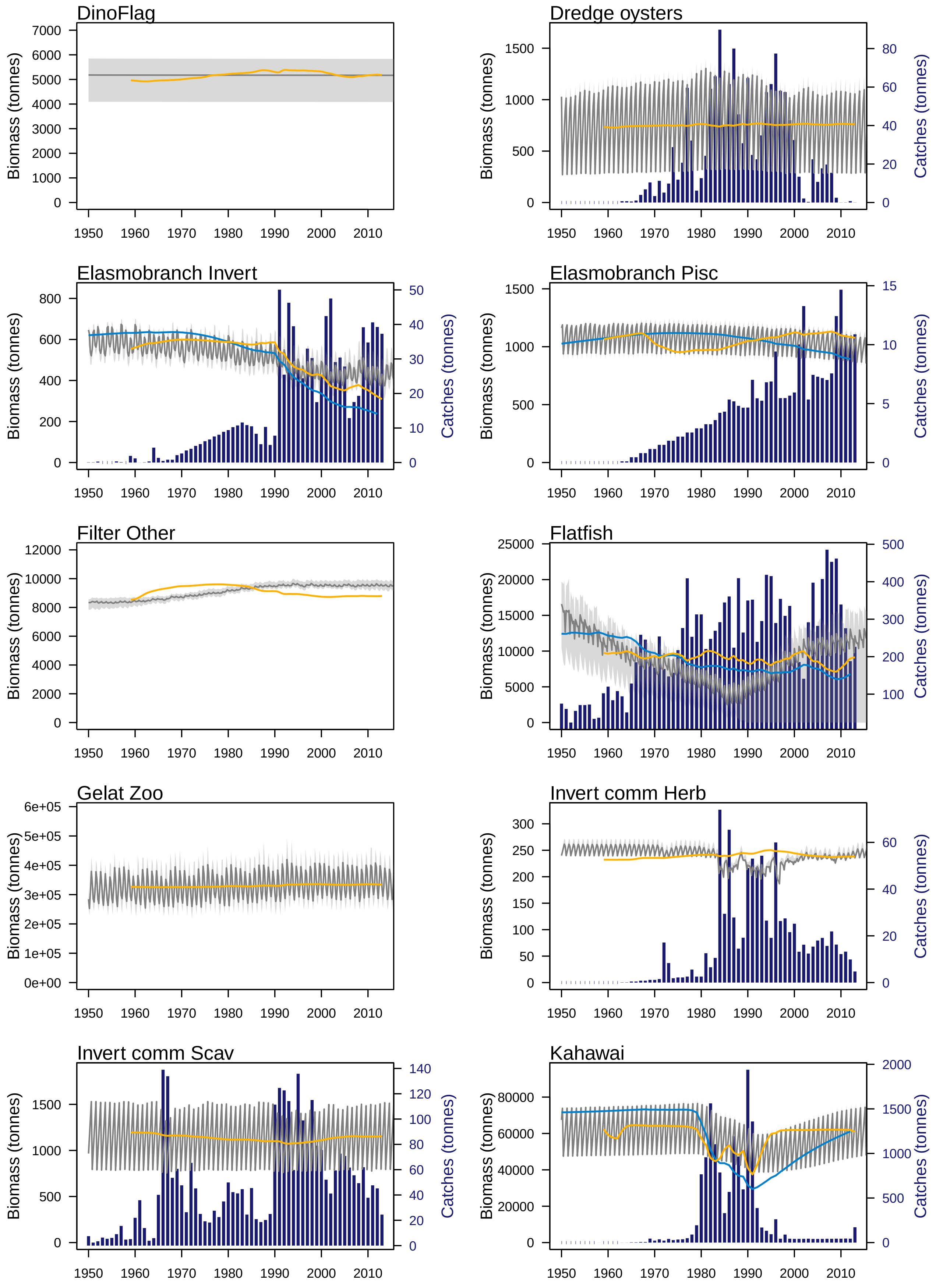
DinoFlag
Dredge oysters
Elasmobranch Invert
Elasmobranch Pisc
Filter Other
Flatfish
Gelat Zoo
Invert comm Herb
Invert comm Scav
Kahawai
Biomass (tonnes)
Catches (tonnes)
1950
1960
1970
1980
1990
2000
2010

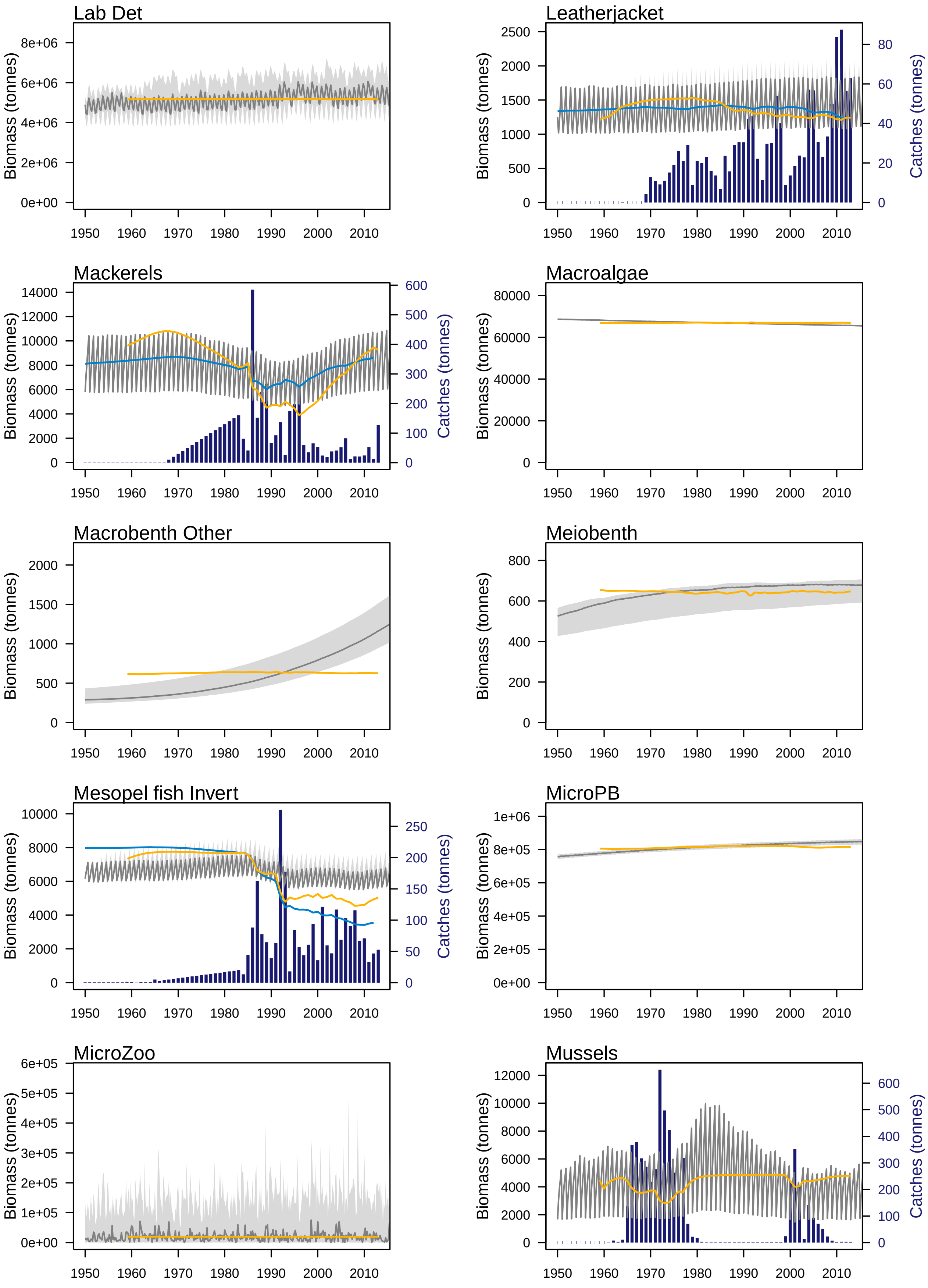
Lab Det
Leatherjacket
Mackerels
Macroalgae
Macrobenth Other
Meiobenth
Mesopel fish Invert
MicroPB
MicroZoo
Mussels
Biomass (tonnes)
Catches (tonnes)
1950 1960 1970 1980 1990 2000 2010

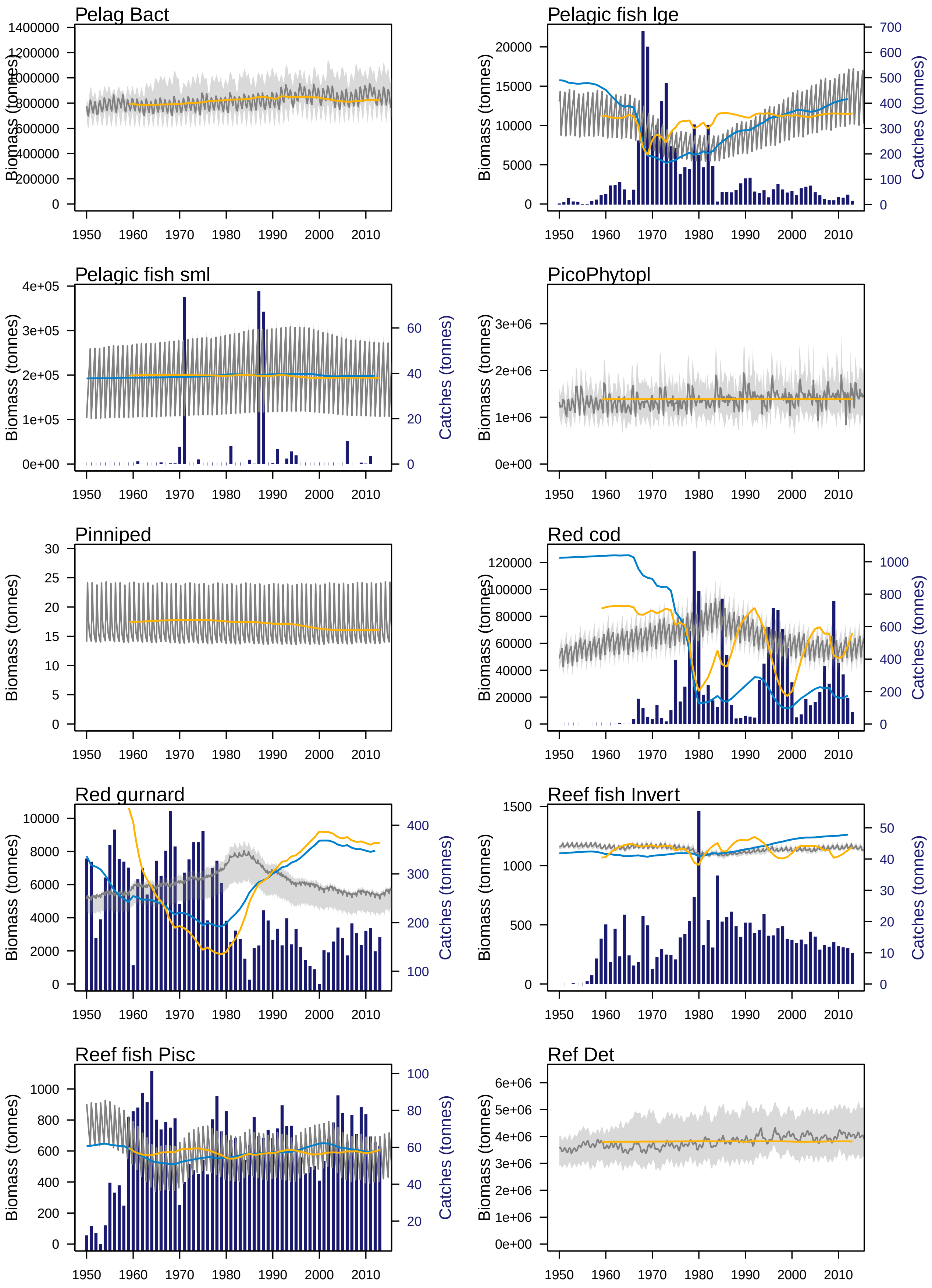
Pelag Bact
Biomass (tonnes)
Pelagic fish lge
Biomass (tonnes)
Catches (tonnes)
Pelagic fish sml
Biomass (tonnes)
Catches (tonnes)
PicoPhytopl
Biomass (tonnes)
Pinniped
Biomass (tonnes)
Red cod
Biomass (tonnes)
Catches (tonnes)
Red gurnard
Biomass (tonnes)
Catches (tonnes)
Reef fish Invert
Biomass (tonnes)
Catches (tonnes)
Reef fish Pisc
Biomass (tonnes)
Catches (tonnes)
Ref Det
Biomass (tonnes)
1950
1960
1970
1980
1990
2000
2010

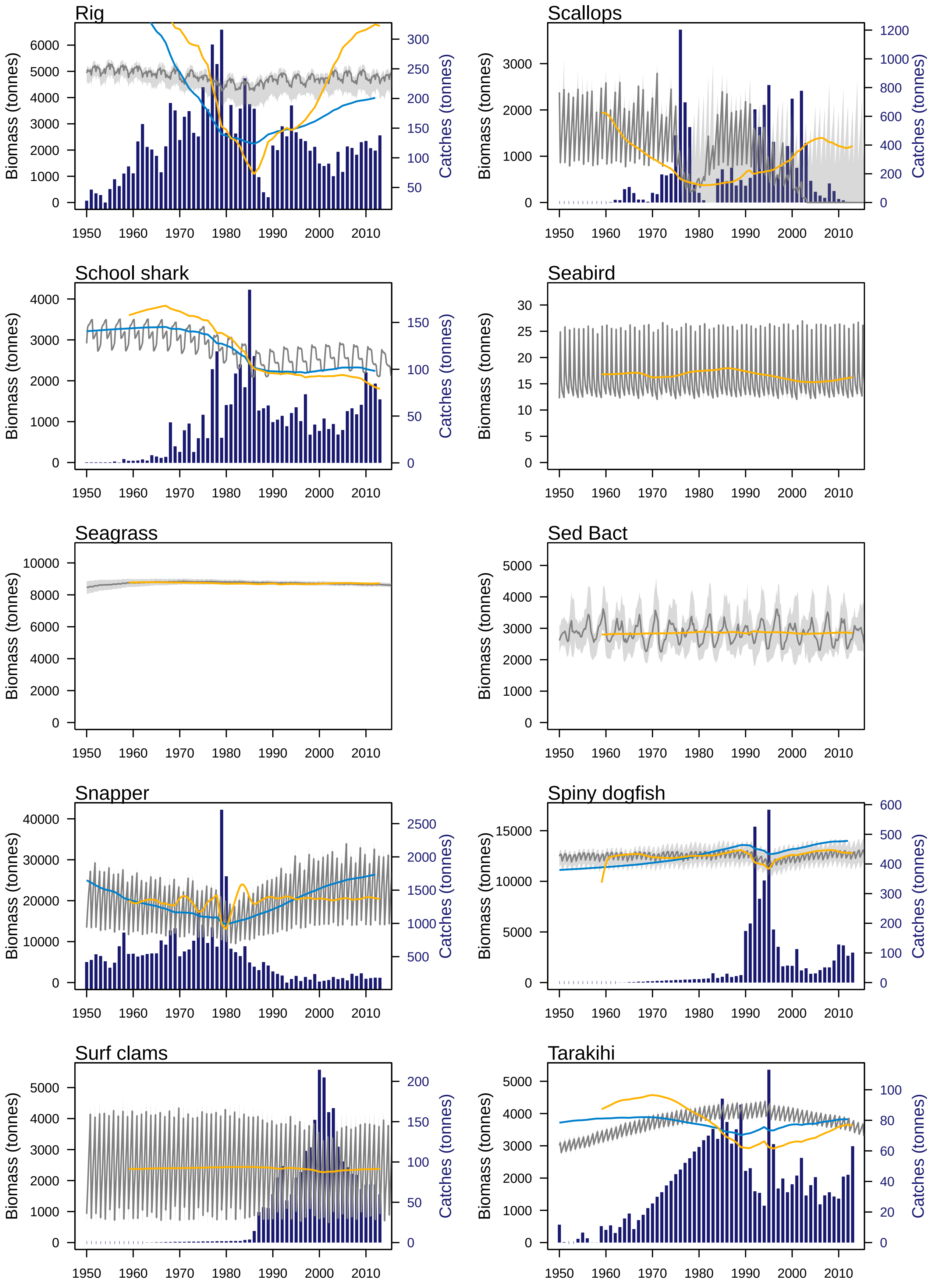
Rig
Scallops
School shark
Seabird
Seagrass
Sed Bact
Snapper
Spiny dogfish
Surf clams
Tarakihi
Biomass (tonnes)
Catches (tonnes)
1950
1960
1970
1980
1990
2000
2010

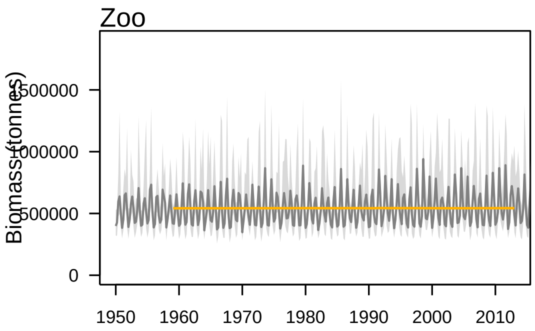

Zoo
Biomass (tonnes)
1500000
1000000
500000
0
1950
1960
1970
1980
1990
2000
2010

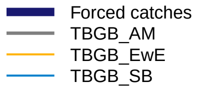

Forced catches
TBGB_AM
TBGB_EwE
TBGB_SB

Supplementary J: Parameters tuned during model calibration

Table 3: Parameters tuned and/or edited during model calibration.

Parameter	Description	General purpose of tuning
pPREY	prey availability	Tuned for realistic realised diets, growth rates and mortality rates
mum (biomass pool species)	Growth of biomass pool species groups	Tuned to provide sufficient food for predators
mQ (biomass pool species)	Quadratic (density dependent)additional mortality	Tuned to limit biomass expansion in cells with less predation pressure
mL (age-structured species groups)	Linear additional mortality	Adjusted to obtain realistic decay curves in the absence of fishing
mum, ht, C (age-structured species, predacse 8)	Maximum growth, handling time and clearance rate used in Holling Type II feeding response	Explored and adjusted, but only with respect to feeding rates and growth rates in the base case
KLP, KUP (age structured species groups)	Upper and lower gape sizes	Explored and adjusted with respect to size-at-age of predator and their prey, and obtaining realistic realised diets
KI (primary producers)	Light saturation	Explored and adjusted with respect to light levels and food required
FSM, FSMG (migratory species)	mortality and growth applied on reentering the model	Adjusted with respect to growth and mortality curves of migrating species groups

1 References

Baird, S., 2011. New zealand fur seals – summary of current knowledge. New Zealand Aquatic Environment and Biodiversity Report 72.

Beentjes, M. P., 2000. Assessment of red cod stocks (rco 3 and rco 7) for 1999. New Zealand Fisheries Assessment Report 2000/25.

Breen, P., 1995. Report on MLS implications for Challenger scallops. Report prepared for the Challenger Scallop Enhancement Company. Unpublished report held by MPI, Wellington, 9p.

Broekhuizen, N., Gurney, W., Jones, A., Bryant, A., 1994. Modelling compensatory growth. Functional Ecology 8(6), 770–782.

Bull, B., Francis, R., Dunn, A., McKenzie, A., Gilbert, D., Smith, M., Bian, R., 2012. CASAL (c++ algorithmic stock assessment laboratory): Casal user manual v2.30–2012/03/21. NIWA Technical Report 135.

Colman, J., 1978. Tagging experiments on the sand flounder, *Rhombosolea plebeia* (Richardson), in Canterbury, New Zealand, 1964 to 1966. Fisheries Research Bulletin (New Zealand) 18.

Drummond, K., Wilson, A., 1993. The biology and purse-seine fishery of kahawai (*Arripis trutta* Bloch and Schneider) from central New Zealand, during 1990/91–1991/92. Central Fisheries Region Internal Report 22 (Unpublished report available from NIWA library, Greta Point, Wellington).

Fisheries New Zealand, 2020. Fisheries Assessment Plenary, May 2020: stock assessments and stock status. Compiled by the Fisheries Science and Information Group, Fisheries New Zealand, Wellington, New Zealand, 1746 p.

Francis, M., Mulligan, K., 1998. Age and growth of New Zealand school shark, *Galeorhinus galeus*. New Zealand Journal of Marine and Freshwater Research 32, 427–440.

Francis, M., Ó Maolagáin, C., 2000. Age, growth and maturity of a New Zealand endemic shark (*Mustelus lenticulatus*) estimated from vertebral bands. Marine and Freshwater Research 51, 35–42.

Francis, M., Ó Maolagáin, C., Stevens, D., 2004. Revised growth, longevity and natural mortality of smooth skate (*Dipturus innominatus*). Final Research Report for Ministry of Fisheries Project MOF2003/01H. (Unpublished report available from Ministry for Primary Industries, Wellington).

Francis, M., Paul, L., 2013. New Zealand inshore finfish and shellfish commercial landings. New Zealand Fisheries Assessment Report 2013/55.

Froese, R., Pauly, D., 2000. FishBase 2000: concepts, design and data sources. ICLARM, Los Baños, Philippines.

Gilbert, D., Sullivan, K., 1994. Stock assessment of snapper for the 1992–93 fishing year. New Zealand Fisheries Assessment Research Document 1994/3.

Hanchet, S., 1986. The distribution and abundance, reproduction, growth and life history characteristics of the spiny dogfish (*Squalus acanthias Linnaeus*) in New Zealand. PhD Thesis, University of Otago, New Zealand.

Hanchet, S., Francis, M., Horn, P., 2001. Age and growth of John dory (*Zeus faber*). New Zealand Fisheries Assessment Report 2001/10.

Horn, P., 1991. Assessment of jack mackerel stocks off the central west coast, New Zealand, for the 1990–91 fishing year. New Zealand Fisheries Assessment Research Document 91/6 (Unpublished report available from NIWA library, Greta Point, Wellington.).

Horn, P., 2001. Validated ageing methods for blue warehou (*Seriolella brama*) and white warehou (*S. caerulea*) in New Zealand waters. Marine and Freshwater Research 52, 297310.

Mackay, K., 2000. Database documentation: trawl. Tech. rep., NIWA Internal Report. URL https://https://marlin.niwa.co.nz/files/sources/CE-1stopshop/DataSystems/Research

Manning, M., Sutton, C., 2007. The composition of the commercial and research giant stargazer (*Kathetostoma giganteum*) catch off the west coast of the South Island (STA 7) during the 2004–05 fishing year. New Zealand Fisheries Assessment Report 36, 36.

Marine Department Annual Report, 1916–1931. [Annual reports presented to the New Zealand Parliament.] Available from the NIWA library, Greta Point, Wellington.

Osborne, T. A., 1999. Nelson Dredge Oyster Stock Assessment. Osborne Research Company Ltd. Client Report ORCO Report 7.

Paul, L., Ó Maolagáin, C., Francis, M., Dunn, A., Francis, R., 2000. Age, growth, mortality, and yield per recruit for butterfish (*Odax pullus*) in Cook Strait, New Zealand. New Zealand Fisheries Assessment Report 2000/6.

Paul, L., Taylor, P., Parkinson, D., 2001. Pilchard (*Sardinops neopilchardus*) biology and fisheries in New Zealand, and a review of pilchard (*Sardinops, Sardina*) biology, fisheries, and research in the main world fisheries. New Zealand Fisheries Assessment Report 2001/37.

Schuckard, R., Melville, D., 2013. Shorebirds of Farewell Spit, Golden Bay and Tasman Bay. Report prepared for Nelson City Council and Tasman District Council. Available at: http://osnz.org.nz/sites/osnz.org.nz/files/Schuckard84.

Stevenson, M., Horn, P., 2004. Growth and age structure of tarakihi (*Nemadactylus macropterus*) off the west coast of the South Island. New Zealand Fisheries Assessment Report 2004/11.

Stevenson, M., MacGibbon, D., 2018. Inshore trawl survey of the west coast South Island and Tasman and Golden Bays, March-April 2017 (KAH1703). New Zealand Fisheries Assessment Report 2018/18.

Sutton, C. P., 1997. Growth parameters, and estimates of mortality for red gurnard (*Chelidonichthys kumu*) from off the east and west coasts of the South Island, New Zealand. New Zealand Fisheries Assessment Research Document 1997/1 (Unpublished report available from NIWA library, Greta Point, Wellington.).

Taylor, R., Barton, K., Wilson, P., Thomas, B., Karl, B., 1995. Population status and breeding of New Zealand fur seals (*Arctocephalus forsteri*) in the Nelson-northern Marlborough region, 1991–94. New Zealand journal of marine and freshwater research 29 (2), 223–234.

Visconti, V., Trip, E., Griffiths, M., Clements, K., 2018. Life-history traits of the leatherjacket *Meuschenia scaber*, a long-lived monacanthid. Journal of fish biology 92 (2), 470–486.

Walsh, C., McKenzie, J., Ó Maolagáin, C., Stevens, D., Tracey, D., 1999. Length and age composition of trevally in commercial landings from TRE 1 and TRE 7, 1997–98. NIWA Technical Report 66.
